# A Retrospective Study of the Evolution of Orthopaedic Injuries in 70 Dressage Horses

**DOI:** 10.3390/ani15121740

**Published:** 2025-06-12

**Authors:** Ana Boado, Danica Pollard, Sue Dyson

**Affiliations:** 1 Independent Researcher, Equine Orthopaedics and Sports Medicine Service, Avenida Salmoral 4, 28492 Madrid, Spain; 2 Universidad Complutense de Madrid, Av. Complutense, Moncloa-Aravaca, 28040 Madrid, Spain; 3 Independent Researcher, Rodham Road, Christchurch, Wisbech PE14 9NU, Cambridgeshire, UK; drdee.pollard@gmail.com; 4 Independent Researcher, Church Road, Market Weston, Diss IP22 2NX, Suffolk, UK; sue.dyson@aol.com

**Keywords:** lameness, poor performance, thoracolumbar, lumbosacroiliac, suspensory desmitis, osteoarthritis

## Abstract

There is limited information about the orthopaedic health of dressage horses. This study aimed to document the orthopaedic status, lesion distribution, and evolution of injuries in 70 horses undergoing a minimum of five in-depth orthopaedic assessments at six-months’ intervals. Warmblood (70.0%) and Iberian (24.3%) breeds predominated. The median lameness grade at initial examination was 2/5 (interquartile range 2,2; range 0,3). Hypermetria (42.9%) or hypermetria and weakness (12.9%) were observed at the initial examination and did not change over time. Metacarpophalangeal/metatarsophalangeal joint region injuries (osteoarthritis or suspensory branch injury), 58.6%, predominated at the initial examination; there was a high proportion of persistent or recurrent injuries (90%). Metacarpal/metatarsal region pain (predominantly suspensory desmitis) had a high prevalence at all examinations (24.3–41.4%), and a high proportion of recurrent injury (90%). There was an increase in spinal pain comparing the initial and final examinations (McNemar’s *p* < 0.001); 21.4% of horses with thoracic region pain subsequently developed lumbosacroiliac region pain. Twenty-six (37.1%) increased work level; nineteen (27.1%) remained at the same level, with 37% competing at Intermediate I or II, under 25 international, or Grand Prix at the final examination, compared with 3% at the initial examination. Serial monitoring and targeted treatment may facilitate horses reaching athletic potential.

## Simple Summary

Seventy dressage horses, mostly Warmblood (70%) and Iberian (24%) breeds, underwent at least five comprehensive orthopaedic examinations at six-month intervals. Outcome was defined as good (returned to the previous level of work or higher) or poor (retired or working at a lower level than baseline). At the initial examination, lameness grade was 0 (0–5 scale) in only three horses; the most frequent lameness grade was 2. The presence of multilimb lameness at the initial examination did not influence the outcome. Horses with grade 0 or 1 lameness at the initial examination had a better outcome than horses with higher grades. More horses that presented initially for routine evaluation (n = 28) had a good outcome (71%) compared with horses with a history of lameness, reduced performance, or swelling (n = 42, 60% with a good outcome). Despite the persistence of lameness over serial examinations in most horses, many were still able to perform and increase their level of training and competition performance, with 61% reaching Prix St Georges level or higher, compared with 30% at the initial examination. Only 22% of horses with hypermetria and weakness had a good outcome. Regular orthopaedic monitoring and targeted treatment may help dressage horses meet their athletic potential.

## 1. Introduction

Orthopaedic problems represent an important economic loss and welfare concern in the equine industry [1]. It has been demonstrated that a high proportion of poorly performing horses have subclinical disorders of the musculoskeletal system [2,3,4,5,6,7].

Lameness is a symptom of an alteration of the normal gait pattern because of a functional or structural disorder in the locomotor system [8]. Horses adapt to lameness with compensatory strategies by altering their gait to reduce forces over injured tissues [8], causing overloading of other structures in ipsilateral and contralateral limbs. The progression and consequences of these compensations over time have not previously been investigated. Horses compensate not only during trot and canter, as previously reported [8], but also at rest and during walk.

Dressage is an Olympic discipline based on close collaboration between a rider and a horse in performing sequences of exercises requiring variable skills and inducing different biomechanical stresses and strains [9]. During competitions, each sequence of exercises a rider and a horse perform receives a score from judges based on predetermined observable criteria. Training of all these exercises is performed by repetition and gradual adaptation of musculoskeletal strength and coordination. The aim is to perform the exercises willingly and harmoniously and without signs of pain or lameness.

Despite the rise in interest in dressage [3] and the increasing value of dressage horses, there are limited publications regarding their injuries [3,10,11]. Risk factors for orthopaedic injuries in dressage horses have been based on owners’ responses to questionnaires [9], with the inherent limitation of the reliability of the information.

As previously reported in human research, “time is the best diagnostician” [12], insights from studies, including repeated observations over time, can help to recognize emerging pictures and anticipate typical trajectories. To the authors’ knowledge, there are limited follow-up studies in equine musculoskeletal diseases/injuries that cover several years. A five-year survival analysis was performed on Icelandic horses with and without radiological evidence of osteoarthritis of the distal hock joints [13]. Several studies have followed the performance of Thoroughbred racehorses with a variety of injuries [14,15,16] or have followed the evolution of specific lesions in the metatarsophalangeal joints of yearling Thoroughbreds entering training over the following two years [17]. Subclinical lesions of the suspensory ligament have been reported in racehorses [18,19] and showjumpers [20]; however, there is limited information about the long-term progression of these injuries and their impact on the development of other injuries. In human athletes in training and competition, it is well-described that lesions may occur at many sites during their careers, and compensatory injuries are frequent [21,22].

Long-term follow-up studies are relatively rare in equine musculoskeletal scientific publications [23,24,25,26], and information has been based on owner-completed questionnaires or records of performance [3,27,28,29,30,31], rather than serial re-examinations, with consequent limitations. There is an absence of knowledge about the progression of injuries in dressage horses.

The objectives of the current study were to identify the presence and to evaluate the progression of orthopaedic injuries in dressage horses through a period of up to five years, and to relate the presence of injury to the horses’ performance outcome. It was hypothesized that 1. the prevalence of spinal pain would increase over time; 2. the presence of lameness in more than one limb at the initial examination would adversely affect the long-term outcome; and 3. there would be no difference in long-term outcome for Iberian and Warmblood horses.

## 2. Materials and Methods

### 2.1. History, Signalment, and Work Level

The clinical records of horses training or competing in dressage between 2009 and 2023 were reviewed. The horses were examined by a single private equine practitioner, an American and European Diplomate in Equine Veterinary Sports Medicine and Rehabilitation (AB). The initial orthopaedic examination was performed either because the owner/rider was concerned that there was a reduction in performance quality, or there was swelling or lameness. Alternatively, an owner requested orthopaedic examination without suspicion of an underlying problem (‘routine’ examination) or, for horses less than five years of age, the owner requested assessment of future performance and potential for sale. Each horse was re-examined on at least four occasions at an interval of approximately 6 months between sequential examinations. Only horses with a minimum of five examinations and comprehensive clinical and imaging data were included.

The level at which a horse was working/competing was classified as 1. young horses (horses up to 7 years of age) and amateurs (riders and horses working below Prix St Georges), 2. Prix St Georges, 3. Intermediate I and II and International classes for riders under 25 years of age (U25), and 4. Grand Prix.

A comprehensive history was obtained at the first examination, comprising age, sex, and breed, reason for examination, specific difficulties the rider had experienced, and any prior history of orthopaedic injuries or observations made at a previous pre-purchase examination. Any other diseases and known surgical interventions were also recorded. At each re-examination, the training/competition level was recorded along with any performance-related problems. Treatments performed by other veterinary teams while training and competing in other countries were also recorded.

### 2.2. Orthopaedic Examinations

All horses were initially examined in a stable to evaluate demeanour, posture, conformation, muscle symmetry, hoof capsule shape and size, and behaviour. Systematic palpation of all four limbs, the cervicothoracic, thoracolumbosacral, and pelvic regions was performed [32]. Range of motion and reaction to passive distal and proximal limb flexion of the forelimbs and hindlimbs were evaluated. Epaxial muscle tone, reaction to palpation, and range of motion of the thoracolumbosacral region were assessed.

Dynamic examination was performed initially on a hard surface in straight lines at the walk and trot, observing from behind, in front, and from the side. Lameness was graded on an ordinal scale of 0–5 (0 = non-lame, 1 = subtle, 2 = mild, 3 = moderate, 4 = severe, 5 = non-weightbearing), under each circumstance in which a horse was examined. Any variations in limb flight or limb placement were recorded. Flexion tests (distal limb flexion in the forelimbs and both distal and proximal limb flexion in the hindlimbs, each for 45 s) were performed in most of the examinations, unless contraindicated by the prior history, a horse’s behaviour, or at an owner’s request. Flexion tests were performed in the least lame limb first. Responses to flexion were recorded as negative, mild, moderate, or severe.

Horses were lunged on a circle of approximately 20 m in diameter on a soft surface and then on a hard surface, when it was safe to do so and with the owner’s consent. The presence of any potentially neurological dysfunction (hypermetria [increased range of motion] [33], weakness) was also recorded.

Ridden examination was performed whenever possible. The rider was asked to work the horse as they would normally do, to include walk, trot, and canter around the periphery of the arena and in circles of 20 m and 10 m diameter, and to perform the movements in which the rider or trainer had previously noted reduced performance.

If the lameness grade was severe when a horse was examined in hand or on the lunge, or deteriorated as the horse trotted, diagnostic imaging was performed, directed by the initial clinical examination.

When appropriate to do so, perineural and intra-articular nerve blocks were performed to determine the source(s) of pain [34]. In horses with lameness in more than one limb, nerve blocks were initiated in the lamest limb, followed by other lame limbs.

### 2.3. Recording of Lameness Grade and Affected Region

Lameness grading was performed for each situation in which a horse was observed in straight lines, on a circle on the left rein, on a circle on the right rein (for both soft and hard surfaces independently), and during ridden exercise on each of the left and right reins. Additional notes were also made about the quality of paces (for example, canter lacked a suspension phase). Lameness was regraded after each diagnostic anaesthetic technique.

Injuries were classified into anatomical regions: foot region (all structures distal to the level of the proximal phalanx), fetlock region (including the digital flexor tendon sheath and suspensory branches), metacarpal/metatarsal region, carpus/tarsus, and proximal limb (humeroradial joint, scapulohumeral joint, femoropatellar, femorotibial and coxofemoral joints, and related soft tissues, for example, the tendon of biceps brachii), and axial skeleton and related soft tissues (‘spinal region’). The spinal region was subdivided into cervical, thoracic, and lumbosacroiliac regions.

### 2.4. Imaging

Diagnostic imaging (radiography and ultrasonography) was directed by the results of the initial clinical assessment and the results of nerve blocks, where applicable. Standard radiographic imaging sequences were acquired [35] with additional views as required, using portable radiographic equipment (Sound Eklin Tru DR LX, Sound-Eklin, Grand Rapids, MI, USA followed by Varex DR, XR PAD 2HR 100uM pixel, Varex imaging, Pioneer Road, Salt Lake City, UT, USA from 2019 onwards). In routine examinations in young horses less than 5 years of age or recently acquired horses, radiographs were either obtained or were available for review from previous pre-purchase examinations. The radiographic protocols for these horses varied but included at least lateromedial images of the front feet and metacarpophalangeal and metatarsophalangeal joints, plantarolateral-dorsomedial oblique images of the tarsi, and lateromedial and caudocranial images of the stifles.

Ultrasonography was performed using a Z One, Zonare Medical Systems, Mountain View, CA, USA, with a 10–14 MHz linear transducer. Transverse and longitudinal images were acquired, with bilateral images always being acquired for comparative purposes. The suspensory ligaments were always evaluated in their entirety (origin, body, and branches). For ‘routine’ examinations, all four suspensory ligaments were assessed. At sequential examinations, any previously identified soft tissue lesions were monitored ultrasonographically. When clinically indicated, skeletal scintigraphy, magnetic resonance imaging (MRI), or computed tomography (CT) was performed. All images were reviewed for the purposes of this study.

### 2.5. Diagnosis

At each examination, the diagnosis was recorded. A recurrent injury is referred to as a lesion that improved structurally, lameness resolved, and the horse was able to resume work, but the same injury redeveloped. A persistent lesion was one that did not resolve (for example, radiological abnormalities consistent with osteoarthritis associated with episodic or continuous lameness). The cause of lameness dictated the treatment and rehabilitation programme, which was tailored for each horse.

The results of five sequential examinations at approximately six-months’ intervals were recorded in a Microsoft Excel (Office 365; Microsoft Corporation, Redmond, WA, USA) spreadsheet. These summarized the identity of the lame limbs, pertinent clinical observations, the most severe grade of lameness at each examination, relevant imaging observations, and the diagnosis(es).

### 2.6. Data Analysis

Data were imported into R Statistical Software (v4.4.2; R Foundation for Statistical Computing, Vienna, Austria. https://www.R-project.org), where all descriptive and inferential statistical analyses were carried out. Horse age at baseline (years) was not normally distributed (Shapiro–Wilk normality test *p* < 0.05), and worst lameness grade (0–5) was an ordinal variable; thus, both were described using median with corresponding interquartile range (IQR) and range. The remainder of the variables relating to horse signalment (breed and sex), dressage level at baseline and at the end of the observation period (young and amateur, Prix St Georges, Intermediate I and II and U25, or Grand Prix), routine access to paddock turnout (yes or no), follow-up outcome compared with baseline (retired, training and competing at a lower level, returned to previous level, or a higher level), recurrence or persistence of injury (yes or no), and the clinical examination were described as proportions and expressed as percentages.

The Pearson’s chi-squared (***Χ*^2^**) test (with Yate’s continuity correction) or Fisher’s exact test (when any one observed frequency in the contingency table ≤ 5) was used to assess the relationship between categorical variables. Namely the relationship between (1) follow-up outcome (poor = retired or training and competing at a lower level than baseline vs. good = returned to previous level or higher level than baseline) and the following variables: single or multilimb lameness, type of examination (routine vs. lame or poor performance or swelling), worst lameness grade (as a categorical variable), presence of neurological gait abnormality (hypermetric, hypermetric and weak, or normal), breed, sex, dressage level started at and dressage level reached; (2) recurrence of injury and the most common site for soft tissue injury; (3) presence of neurological gait abnormality and dressage level reached. Cramér’s V was additionally calculated to estimate the effect size (ES) measure for the ***Χ^2^*** test. The strength of association for a ***Χ^2^*** test with degrees of freedom (df) = 1 was interpreted as weak if ES < 0.30, moderate if 0.30 < ES < 0.50, and strong if ES ≥ 0.50. The strength of association for a ***Χ^2^*** test with df = 2, as suggested by Cohen [36], was interpreted as weak if ES < 0.21, moderate if 0.21 < ES < 0.35, and strong if ES ≥ 0.35. For categorical variables with more than two levels and where the ***Χ^2^*** or Fisher’s exact test *p* < 0.05, pairwise comparisons were carried out post hoc to identify which levels were different, including the Bonferroni adjustment for multiple comparisons for *p*-values. McNemar’s test (including continuity correction) was used to identify a difference in the proportion of spinal injury between the first and last examinations.

The Kruskal–Wallis rank sum test was used to assess the relationship between follow-up outcome and worst lameness grade and horse age. The eta-squared measure was also calculated to estimate the ES based on the H-statistic [36] with ES < 0.06 considered weak, 0.06 < ES < 0.14 considered moderate, and ≥ 0.14 considered strong.

The two-sample test for equality of proportions (without continuity correction) based on the Z-test was used to compare the proportion of left forelimb and right forelimb lameness and left hindlimb and right hindlimb lameness at each of the five examination timepoints.

Due to the relatively small sample size and the exploratory nature of the study, significance testing was not adjusted for multiple comparisons [37]. Although a significance threshold of *p* < 0.05 was used, large observed differences may be clinically relevant and thus clinically rather than statistically significant [38]. Hence, we have presented effect sizes and 95% confidence intervals (CIs) where applicable, alongside *p*-values, to allow readers to assess the range of results and judge their clinical relevance [39].

## 3. Results

### 3.1. Horse Signalment and Work Level

There were 70 horses in the study with a median age of 7 years (IQR 3,10; range 3,14 years) at the first examination. Breeds compromised 49 (70.0%) Warmbloods, 17 (24.3%) Iberian breeds (Pure Spanish breed and Lusitano), and 4 crossbreeds (5.7%). There were 51 (72.9%) geldings, 15 stallions (21.4%), and 4 mares (5.7%). Fifty-eight horses (67.1%) routinely had paddock turnout.

**Figure 1:**
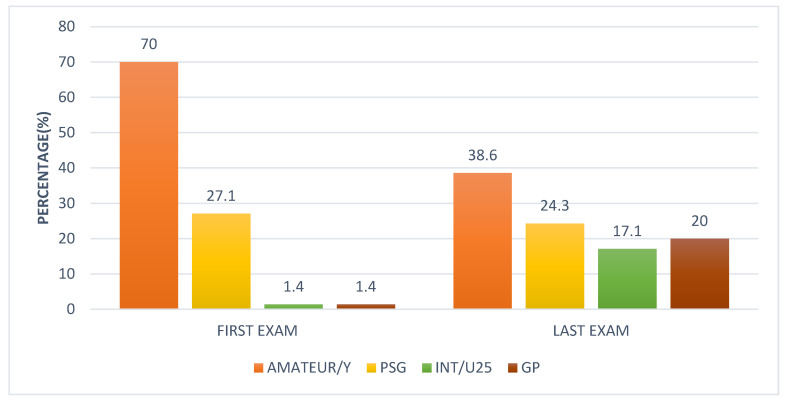
Comparison of work level of 70 dressage horses at the first examination and at the final examination after between 2.5 and 5 years. The initial examination was performed either because the owner/rider was concerned that there was a swelling, lameness, or reduction in performance quality, or the owner requested a routine evaluation, or, for horses less than five years of age, an assessment of future performance or potential for sale. Each horse was re-examined on at least four occasions with a minimum interval of 6 months between sequential examinations. Amateur/Y = Amateur/young horses training and competing below Prix St Georges; PSG = Prix St Georges; Int/U25 = Intermediate I or II/International Under 25 years; GP = Grand Prix.

At the first examination, there were 49 amateur and young horses (70.0%), 19 horses (27.1%) training/competing at Prix St Georges, 1 horse at Intermediate I and/or II and U25 (1.4%), and 1 (1.4%) at Grand Prix. At the final examination, 27 horses (38.6%) were young horses or amateur levels, 17 (24.3%) at Prix St Georges level, 14 horses (20.0%) were working at Grand Prix, and 12 (17.1%) at Intermediate I or II or U25 (Figure 1).

### 3.2. Orthopaedic Examination

The reason for the initial examination was because of swelling, lameness, or decreased performance in 42 horses (60.0%) and a request for a routine assessment in 28 horses (40.0%). Lameness grade was 0 in only 3 (4.3%) horses, grade 1/5 in 8 (11.4%) horses, grade 2/5 in 44 (62.9%) horses, and grade 3/5 in 15 (21.4%) horses. Median lameness grade was 2/5 (IQR 2,2; range 0,3). Grades 2 and 3 were the most common, present in 59 (84.3%) horses.

Thirty-four of sixty-seven lame horses (50.7%) exhibited lameness in straight lines at the initial examination. Fifty-three horses (79.1%) showed lameness on the lunge, and twenty-nine horses (43.3%) were lame when ridden. Thirty-nine of seventy horses (55.6%) had at least one positive flexion test.

**Figure 2:**
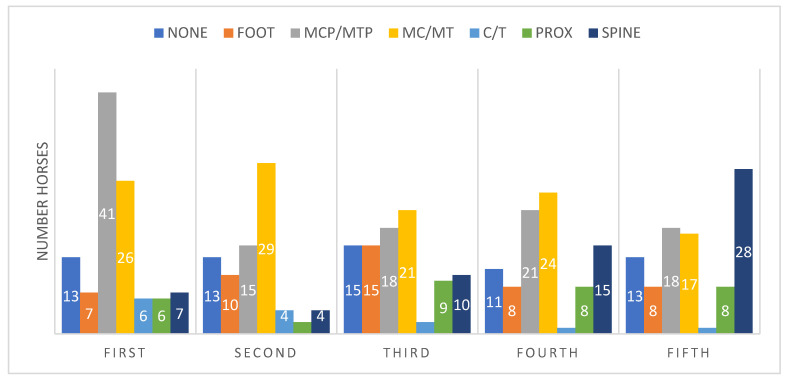
Anatomical regions of injury in 70 dressage horses examined sequentially at six-months’ intervals for five examinations. The initial examination was performed either because the owner/rider was concerned that there was a reduction in performance quality, lameness, or swelling, or as a routine examination, or, for horses less than five years of age, the owner requested an assessment of future performance or potential for sale. Each bar represents the number of horses per anatomical region, with some horses having more than one injury site. None = no abnormality detected. MCP = Metacarpophalangeal joint region, MTP = Metatarsophalangeal joint region. Mc/MT Metacarpal, Metatarsal region, C carpus, T tarsus, PROX = proximal to carpus or tarsus, Spine = cervical, thoracic, and lumbosacroiliac regions.

Multilimb lameness was detected in 49 horses (70.0%), whereas single limb lameness was present in 21 (30.0%) horses at any of the examinations. The anatomical regions of injury at each examination are summarized in Figure 2. The proportion of horses at each examination in which no specific active injury was identified ranged from 17% to 29%. Metacarpophalangeal/metatarsophalangeal joint region injuries (58.6%) predominated at the initial examination. The prevalence of spinal injuries was 10.0% at the initial examinations, rising to 40.0% at the fifth examination. There was a significant increase in the proportion of spinal injuries over time when comparing the first and final examinations (McNemar’s *p* < 0.001). Metacarpal/metatarsal region injuries had a high prevalence at all examinations, ranging from 24.3% to 41.4%.

At the initial examination, predominant left forelimb lameness was detected in 28 (40%) horses, right forelimb in 22 (31%), left hindlimb in 22 (16%), and right hindlimb in 11 (16%) of the horses. The sum exceeds 70 horses because some horses with lameness in more than one limb had the same lameness grade in more than one limb. When considering all examinations, predominant left forelimb lameness was detected in 19 horses (13.3%), right forelimb lameness in 11 (7.7%), left hindlimb lameness in 11 (7.7%), and right hindlimb lameness in 7 (4.9%).

### 3.3. Neurological Examination

Thirty horses (42.9%) had a hypermetric gait at the first examination, whereas nine horses (12.9%) showed both hypermetria and weakness. Minimal change was observed at subsequent examinations.

### 3.4. Evolution of Lameness over Time

**Figure 3:**
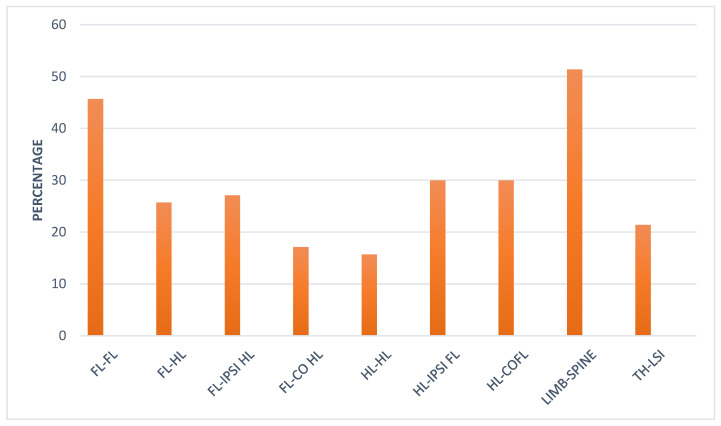
Evolution of injury in 70 dressage horses examined sequentially at six-months’ intervals for five examinations. The initial examination was performed either because the owner/rider was concerned that there was a reduction in performance quality, lameness, or swelling, or as a routine examination, or, for horses less than five years of age, the owner requested an assessment of future performance or potential for sale. The bars represent the percentage of horses that changed from predominant injury in one limb to another limb, or from a limb to the spinal region, or from the thoracic spinal region to the lumbosacroiliac region. FL = Forelimb, HL = Hindlimb. IPSI = ipsilateral, CO = Contralateral, TH = Thoracic, LSI = Lumbosacroiliac.

Thirty-two horses (46.0%) that initially had unilateral forelimb lameness developed contralateral forelimb lameness (Figure 3). Nineteen horses (27.0%) with forelimb lameness developed ipsilateral hindlimb lameness. Twelve horses (17.1%) with forelimb lameness developed contralateral hindlimb lameness. A difference in the proportion of horses with forelimb lameness that developed ipsilateral hindlimb lameness and horses with forelimb lameness that developed contralateral hindlimb lameness was not identified (two-sample test for equality of proportions, *p* = 0.154).

Of the horses that initially had hindlimb lameness, 11 (15.7%) developed contralateral hindlimb lameness. Twenty-one horses (30.0%) with initial hindlimb lameness developed ipsilateral forelimb lameness, and twenty-one (30.0%) developed contralateral forelimb lameness.

Thirty-eight horses (51.4%) that initially showed lameness in any limb subsequently developed spinal pain (Figure 3). Fifteen horses (21.4%) with thoracic region pain subsequently developed lumbosacroiliac region pain.

The difference in the proportion of left and right forelimb lameness and left and right hindlimb lameness was assessed at each examination time point (Table 1). No significant differences in the proportion of forelimb or hindlimb lameness were identified.

The original injury, diagnosed at the first examination, persisted throughout the study in 40 horses (57.1%). Thirty-two horses (54.0%) that had an injury in one limb developed another injury site in the same limb. Recurrence of injury occurred in 58 horses (82.9%).

### 3.5. Outcome

During the study period, 26 horses (37.1%) increased the level of work, and 19 horses (27.1%) remained at the same work level. Sixteen horses (22.9%) decreased the level of work, and nine (12.9%) horses were retired because of orthopaedic injury. Overall, 45 horses (64.3%) had a good outcome, whereas 25 horses (35.7%) had a poor outcome.

**Figure 4:**
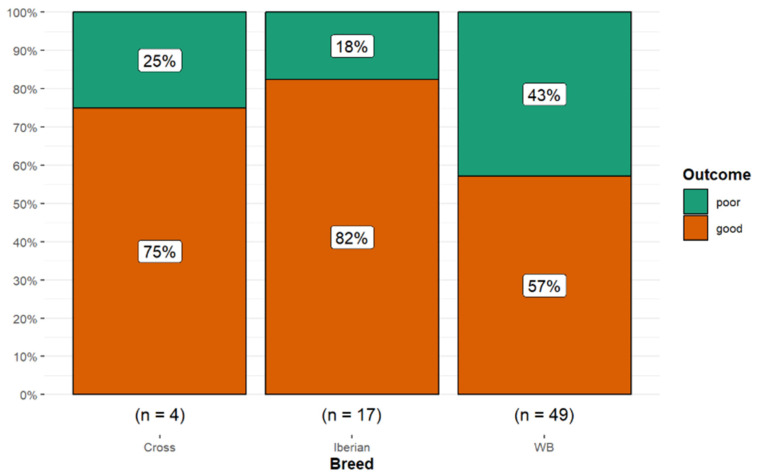
Long-term outcome for 70 dressage horses examined sequentially at six-months’ intervals for five examinations. The initial examination was performed either because the owner/rider was concerned that there was a reduction in performance quality, lameness, or swelling, or as a routine examination, or, for horses less than five years of age, the owner requested an assessment of future performance or potential for sale. A good outcome (orange) was defined as returning to work at the same level or higher; a poor outcome (green) was defined as a decline in work level or retirement. A higher proportion of Iberian and crossbreed horses had a good outcome compared with Warmblood horses.

There were no significant associations between age (Kruskal–Wallis *p* = 1.00; eta-squared = −0.01), breed (Fisher’s exact *p* = 0.139; Cramér’s V = 0.16, 95% CI 0.00, 0.41), sex (Fisher’s exact *p* = 0.677; Cramér’s V = 0.00, 95% CI 0.00, 0.27), work level at the first examination (***Χ*^2^** = 0.00, *p* = 1.00; Cramér’s V = 0.00, 95% CI 0.00, 0.22), or access to paddock turnout (***Χ^2^*** = 0.83, *p* = 0.363; Cramér’s V = 0.07, 95% CI 0.00, 0.36), and the final outcome. However, 14 of 17 Iberian horses (82.4%) and 3 of 4 crossbreeds (75.0%) had a good outcome compared with only 28 of 49 Warmbloods (57.1%) (Figure 4).

Of the horses with paddock access prior to the first examination, 59.6% (n = 28/47) had a good outcome compared with 73.9% (n = 17/23) of horses with no paddock turnout.

Horses undergoing ‘routine’ examinations, without suspicion of a problem or were initially presented at less than 5 years of age because the owner requested an assessment of future performance or potential for sale, had a better outcome (71.4%) than those that were examined because of a decline in performance, lameness, or swelling at the time of the initial examination (59.5%). However, this difference was not statistically significant (***Χ^2^*** = 0.58, *p* = 0.445; Cramér’s V = 0.02, 95% CI 0.00, 0.34).

A similar proportion of horses (n = 14/21, 66.7%) with a single limb lameness at the initial examination had a good outcome as those with multilimb lameness (n = 31/49, 63.3%).

**Figure 5:**
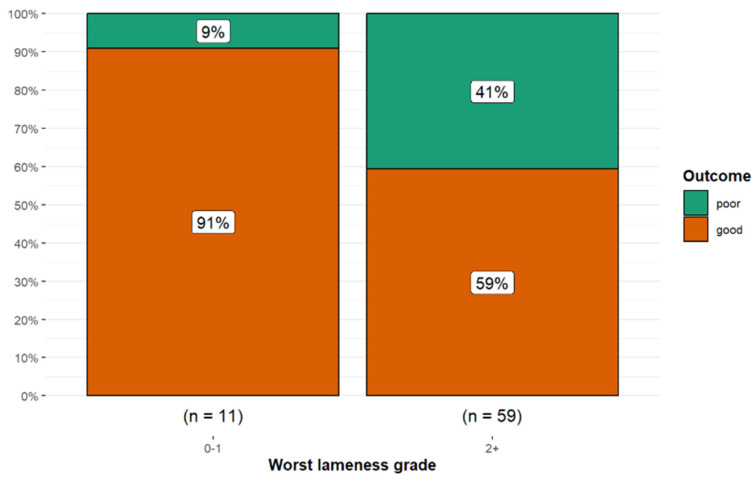
Long-term outcome for 70 dressage horses examined sequentially at six-months’ intervals for five examinations. The initial examination was performed either because the owner/rider was concerned that there was a reduction in performance quality, swelling, or lameness, or as a routine examination, or, for horses less than five years of age, the owner requested an assessment of future performance or potential for sale. A good outcome (orange) was defined as returning to work at the same level or higher; a poor outcome (green) was defined as a decline in work level or retirement. Horses with grade 0 or 1/5 lameness had a better outcome than horses with grade ≥ 2/5 lameness.

There was no significant association between the highest lameness grade and outcome (Kruskal–Wallis *p* = 0.063, eta-squared = 0.04). However, 10 of 11 horses (90.9%) with a Grade 0 or 1/5 lameness at the initial examination had a good outcome compared with only 35/59 horses (59.3%) with grade ≥ 2/5 lameness (Fisher’s exact *p* = 0.083; Cramér’s V = 0.21, 95% CI 0.00, 0.46) (Figure 5).

**Figure 6:**
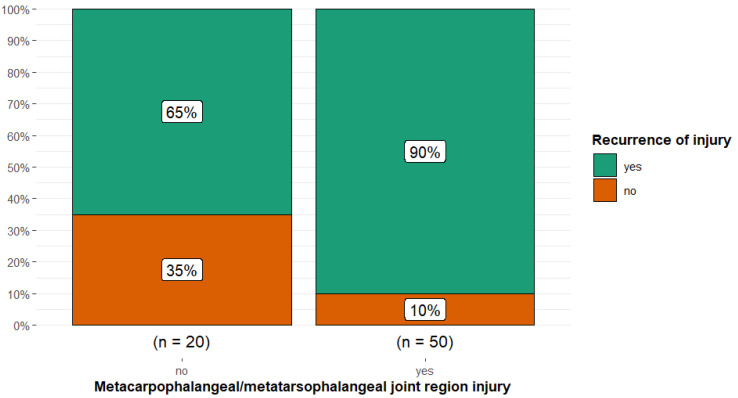
Injury reoccurrence in 70 dressage horses examined sequentially at six-months’ intervals for five examinations, comparing horses without or with metacarpophalangeal or metatarsophalangeal joint region injuries. The initial examination was performed either because the owner/rider was concerned that there was swelling, a reduction in performance quality, or lameness, or as a routine examination, or, for horses less than five years of age, the owner requested an assessment of future performance or potential for sale. There was a higher proportion of persistent or recurrent injury (green) in horses with metacarpophalangeal or metatarsophalangeal region injury (90%) compared with other anatomical sites of injury combined (65%).

There was a higher proportion of persistent or recurrent injury in horses with metacarpophalangeal or metatarsophalangeal joint region injury (90.0%) compared with other anatomical sites of injury (65.0%) (Fisher’s exact *p* = 0.03; Cramér’s V = 0.28, 95% CI 0.00, 0.52) (Figure 6).

**Figure 7:**
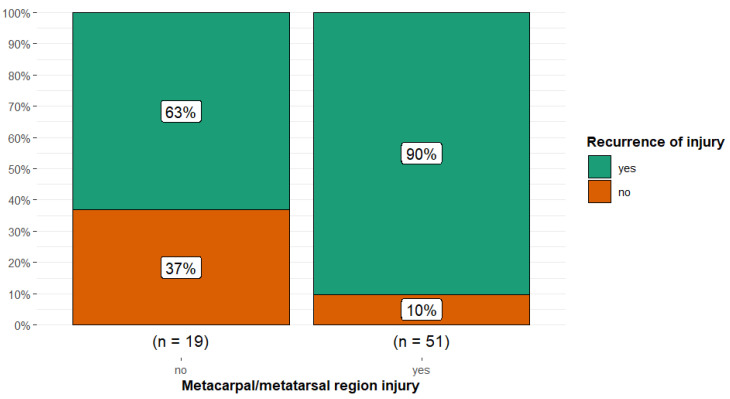
Injury recurrence in 70 dressage horses examined sequentially at six-months’ intervals for five examinations, comparing horses without and with metacarpal or metatarsal region injuries. The initial examination was performed either because the owner/rider was concerned that there was a swelling, reduction in performance quality, or lameness, or as a routine examination, or, for horses less than five years of age, the owner requested an assessment of future performance or potential for sale. There was a higher proportion of recurrent injury (green) in horses with metacarpal or metatarsal region injury (90%) compared with all other anatomical sites of injury combined (63%).

There was a higher proportion of recurrent injury in horses with metacarpal or metatarsal region injuries (90.2%) compared with horses with injuries in all other anatomical regions combined (63.2%) (Fisher’s exact *p* = 0.013; Cramér’s V = 0.30, 95% CI 0.00, 0.54) (Figure 7).

**Figure 8:**
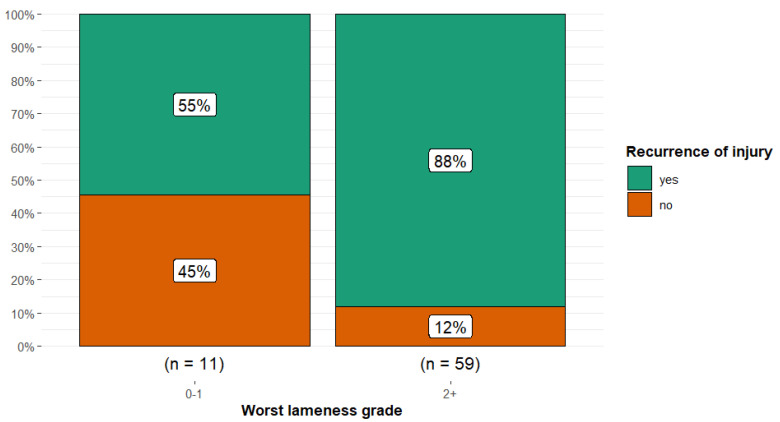
Injury recurrence in 70 dressage horses examined sequentially at six-months’ intervals for five examinations, comparing horses with the highest lameness grades at initial examination of 0–1/5 and those with lameness grades ≥ 2/5. The initial examination was performed either because the owner/rider was concerned that there was a swelling, reduction in performance quality, or lameness, or as a routine examination, or, for horses less than five years of age, the owner requested an assessment of future performance or potential for sale. There was a higher proportion of recurrent injury (green) in horses with higher lameness grades (88%) compared to horses with lower lameness grades (55%).

There was an association between the highest lameness grade during the initial examination and the recurrence of injury (Fisher’s exact *p* = 0.017; Cramér’s V = 0.30, 95% CI 0.00, 0.55) with horses that had lameness grades ≥ 2/5 more likely to have recurrence of injury compared to horses with lameness grades of 0 or 1/5 (Figure 8). There was no association between the number of lame limbs and outcome.

**Figure 9:**
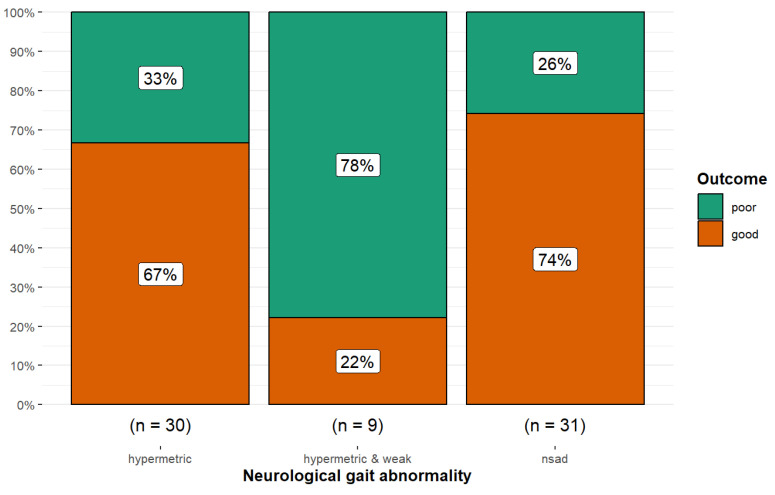
Long-term outcome of 70 dressage horses examined sequentially at six-months’ intervals for five examinations. The initial examination was performed either because the owner/rider was concerned that there was a reduction in performance quality, swelling, or lameness, or as a routine examination, or, for horses less than five years of age, the owner requested an assessment of future performance or potential for sale. A good outcome (orange) was defined as returning to work at the same level or higher; a poor outcome (green) was defined as a decline in work level or retirement. Horses with no neurological gait abnormality or hypermetria were more likely to have a good outcome than horses with hypermetria and weakness (*p* = 0.017). nsad: no significant neurological gait abnormality detected.

There was an overall difference in the proportion of good and poor outcomes between the three categories of neurological gait abnormality (Fisher’s exact *p* = 0.017; Cramér’s V = 0.30, 95% CI 0.00, 0.54). Horses with hypermetric and weak gaits were more likely to have a poor outcome (*p* = 0.029) compared with both horses with no neurological gait abnormality or those with a hypermetric gait (Figure 9).

**Figure 10:**
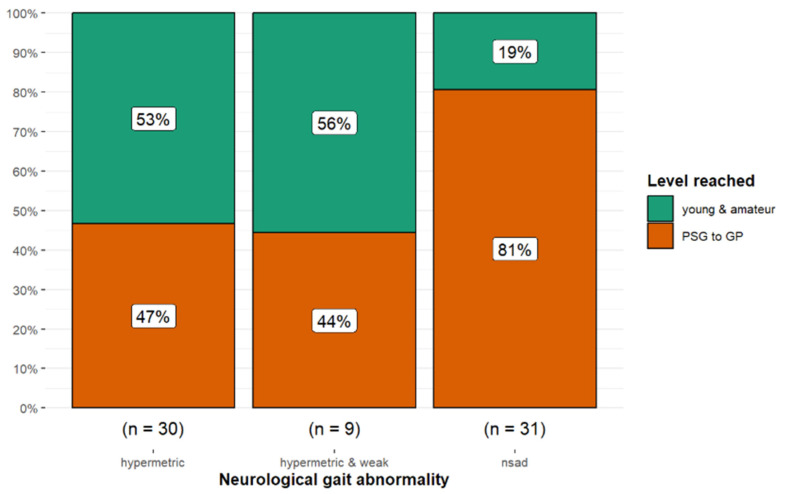
Long-term outcome of 70 dressage horses examined sequentially at six-months’ intervals for five examinations. The initial examination was performed either because the owner/rider was concerned that there was a swelling, reduction in performance quality, or lameness, or as a routine examination, or, for horses less than five years of age, the owner requested an assessment of future performance or potential for sale. Eighty-one percent of horses with no neurological gait abnormality reached Prix St Georges level or higher compared with only 47% and 44% of horses that were hypermetric or hypermetric and weak, respectively. nsad: no significant neurological gait abnormality detected.

There was also a significant difference in work level reached depending on the presence or absence of a potentially neurologically-mediated gait abnormality (Fisher’s exact *p* = 0.011; Cramér’s V = 0.31, 95% CI 0.00, 0.55). Horses with neurologically normal gaits were more likely to reach Prix St Georges level or above (*p* = 0.019) compared with hypermetric horses or those that had hypermetric and weak gaits (Figure 10).

## 4. Discussion

### 4.1. Results Related to Hypotheses

This unique longitudinal study followed the orthopaedic status and career progression of a cohort of 70 dressage horses examined at six-months’ intervals for a total of at least five examinations. In accordance with the first hypothesis, the prevalence of spinal region pain increased over time. However, contrary to the second hypothesis, the presence of lameness in more than one limb at the initial examination did not influence the long-term outcome. Finally, although there was no statistical difference in outcome between Warmblood horses compared with Iberian breeds, Iberian horses had a superior outcome, and this finding may be of biological significance.

#### 4.1.1. Progression from Lameness to Spinal Pain

The association between lameness and thoracolumbar epaxial muscle hypertonicity and pain is well recognized [40]. Most horses were lame at the initial examination, and it is well-documented that reduced range of motion of the thoracolumbosacral region is an adaptation to lameness [41,42]. Abolition of lameness by diagnostic anaesthesia resulted in an increased range of motion of the thoracolumbosacral region [43]. Reduced range of motion of the thoracolumbosacral region will result in muscle atrophy, or in younger horses, a failure to develop appropriate musculature, and likely reduced stability of the vertebral column, predisposing to injury and pain.

In dressage horses working at Prix St Georges level and above, much of the trot work is performed sitting, which alters loading of the thoracic region and thoracolumbar movement [44]. In sitting trot compared with rising trot, a horse with primary back pain often adapts by further reducing the movement of the thoracolumbosacral region [45,46]. When comparing thoracolumbosacral movement in hand compared with ridden exercise in sitting trot and canter in 10 elite dressage horses, movement in the caudal thoracic and lumbar regions altered [47]. The functional long-term consequences of this remain unknown. Moreover, in the lateral movements required in upper-level dressage horses, there are increased rotational movements of the thoracic region, changing force distribution [48]. It is recommended that riders should be encouraged to increase the proportion of work in rising trot versus sitting trot and in a two-point position rather than a three-point position in canter.

In a study of 26 dressage riders in sitting trot, no riders were able to perform anterior or posterior pelvic tilt whilst seated on a ball without demonstrating mild or major compensations and this was reflected when riding by their asymmetry of posture, orientation of the trunk, and inability to move synchronously with the horse [49]. This will inevitably potentially influence loading and motion of the equine thoracolumbar region.

A relationship between thoracolumbar pain and lameness in dressage horses has been previously reported in a questionnaire-based study of 2032 horses [3]. The occurrence of a back problem in the previous two years was strongly associated with lameness over the same period (OR = 12.6; 95% CI = 5.8–27.5; *p* < 0.001).

The progressive increase in spinal pain over sequential examinations in the current study highlights that careful clinical appraisal of the thoracolumbar region, saddle-fit for horse and rider, and rider position should be integral parts of the routine evaluations of dressage horses. Rider morphology and size (height and weight) and fit of the saddle for both horse and rider are critical for optimal force distribution in the thoracic region [50], and if this is not ideal, then epaxial muscle function is compromised, which may have long-term deleterious consequences [51]. The importance of optimal saddle fit for the horse was emphasized by the rapid increase in thoracic dimensions within two months after improved saddle fit [52].

#### 4.1.2. Development of Lumbosacroiliac Joint Region Pain

It was also observed in the current study that more than 20% of horses with thoracic region pain subsequently developed lumbosacroiliac joint region pain. In a United Kingdom-based study of 296 horses with lumbosacroiliac joint region pain, more than one-third (35%) were used for dressage [53], suggesting that dressage horses may be predisposed. Sports horses with thoracolumbar pain had decreased range of dorsoventral flexion and extension at the walk and trot in addition to reduced axial rotation and increased lateral bend at the walk when compared with asymptomatic sports horses [54]. These gait adaptations could result in abnormal loading of the lumbosacroiliac joint region, resulting in pain. Of 64 horses with lumbosacral or lumbar 5–6 symphysis disease, 17% were dressage horses [55]. These observations highlight the importance of managing lameness and thoracolumbar region pain to prevent the sequential development of additional problems.

#### 4.1.3. Multilimb Lameness and Outcome

The presence of lameness in more than one limb at the initial examination did not influence the long-term outcome. This may reflect the high prevalence of lameness from the outset, with 96% of horses showing lameness, recognized through the thoroughness of the examination, and the frequent progression to lameness in other limbs and/or the development of thoracolumbosacral region pain. Although hindlimb lameness can manifest by a head nod and forelimb lameness may induce hindlimb asymmetry (so-called compensatory lameness), in this study, each lameness was abolished by diagnostic anesthesia of the lame limbs and was not considered to be compensatory. Many skilled dressage riders can make subtle adjustments to rebalance horses and effectively inadvertently conceal lameness. This is reflected by the fact that in the current study, at the initial examination of the lame horses, 51% and 79% were lame in hand and on the lunge, respectively, whereas only 43% were lame when ridden. In contrast, in a mixed population of 57 dressage horses and showjumpers considered to be working comfortably, 26% and 41% were lame in hand or on the lunge, respectively, whereas 47% were lame when ridden [56]. However, when the gait of 19 dressage horses, working at least at Prix St Georges level and considered to be non-lame by subjective analysis, was compared objectively during in hand trot and ridden exercise at collected and extended trot (sitting), greater asymmetries of head, withers, and pelvis movement were observed during ridden exercise [57]. The authors suggested that a rider created increased hindlimb impulsion and vertical range of motion when lowering the pelvis and elevating the withers, thereby exacerbating inherent asymmetries in muscular strength. However, the effect of a rider on the gait of a lame horse may differ according to the presence of forelimb or hindlimb lameness, the skill of the rider, sitting or rising trot, and the work level of the horse [58,59,60,61].

The correct training of dressage horses develops musculoskeletal strength and coordination and increased muscle mass, providing axial skeleton stability, which may help to conceal lameness when ridden. Moreover, dressage horses are generally ridden on artificial surfaces, which potentially provide more cushioning than grass, which may enable horses to work reasonably comfortably, despite lameness.

#### 4.1.4. Breed and Outcome

Iberian horses had a non-significant superior long-term outcome compared with Warmblood horses. It is difficult to determine whether this is a true breed difference or reflects the selection of dressage horses by riders and trainers. The Iberian horses were bred in Spain and Portugal, whereas most Warmbloods were imported. Horses are commonly subjected to selection procedures in each country, and the superior horses remain in their native countries. This may reflect movement quality, trainability, and possibly inherent soundness.

There are also differences in gait and conformation between the breeds, which may influence injury risk and longevity [62,63,64]. The more animated gait characteristics of modern Warmblood horses [65] compared with Iberian horses may result in asymmetry of movement being more obvious and therefore career-limiting. The temperaments of the horses and their ability to continue in work despite discomfort may also be of relevance. In any future study, it would be interesting to apply the Ridden Horse Pain Ethogram (RHpE) [66] to assess the behavioral responses to pain in Iberian and Warmblood horses.

### 4.2. Sex Distribution

In this study, there were only 6% of mares, which contrasts with most sports horse studies in which mares often represent up to 50% of the population. In a study of 263 elite showjumpers [67], a convenience sample from four European countries, 46% were mares. Sex did not influence days lost to training. In a Swedish study based on insurance data from more than 138,500 horses, approximately 50% were mares [68]. The reason for the small proportion of mares in the current study is unclear and may reflect a selection bias, even though there are mares that are ranked highly based on performance in Fédération Equestre Internationale (FEI) regulated competitions [69]. The proportion of mares is similar to that described for elite dressage horses (7%) in a previous United Kingdom-based study [10], in contrast with 41% of mares participating in elite showjumping. Overall, among dressage horses registered with the FEI, male horses predominate (87%) [69].

### 4.3. Distribution of the Initially Lame Limb

In the current study, at the initial examination, a higher proportion of horses had left forelimb lameness compared with right forelimb lameness, although the difference was not significant, whereas the prevalences of left hindlimb and right hindlimb lameness were identical. Throughout the study, no left and right limb differences occurred. Most dressage horses are likely to be worked to a similar extent on the left and right reins [70], so a left-right difference in affected limbs would not be anticipated.

#### 4.3.1. Lameness Grading

The median lameness grade was 2/5, although lameness was either not acknowledged or recognized prior to the orthopaedic examination. This is a relevant observation because dressage horses are judged on the quality of their gaits. Some of these horses were not lame when ridden; however, they may have had limitations in their training and decreased scores during competition because of pain, which might only be revealed during an in-depth orthopaedic examination. A previous study demonstrated that high-level trainers failed to recognize hindlimb lameness in 53% of horses when ridden [71]. This suggests that frequent veterinary assessments of dressage horses from a young age might be beneficial for both increasing their quality of performance and extending their careers. This is supported by the observation in the current study that those horses that were presented for routine assessment, or, at less than 5 years of age, for assessment of future performance, had a non-significant superior outcome compared with those examined for swelling, lameness, or poor performance. This difference may be of biological significance. Although there was no association with lameness grade and outcome, 91% of horses with a lameness grade of 0 or 1/5 at the initial examination had a good outcome compared with only 59% of horses with higher grades.

#### 4.3.2. Evolution of Lameness

There was a variety of patterns of evolution of lameness over time. The most common progression was from forelimb to forelimb, followed by hindlimb to forelimb (both ipsilateral and contralateral) and forelimb to ipsilateral hindlimb. Patterns of compensation and biomechanical effects of mild lameness have been studied at the walk and the trot [3]. With reduced loading of the lame forelimb, there was an increased loading of the contralateral forelimb, and to a lesser extent, of the hindlimbs at walk. At trot, stride frequency increases, and ground reaction forces in the lame forelimb decrease, with increasing force on the contralateral forelimb and ipsilateral hindlimb. With unilateral hindlimb lameness, the vertical impulse increased in the contralateral hindlimb. These compensatory mechanisms emphasize the need for early recognition of lameness to optimize the potential success of treatment and management, to reduce the risk of developing lameness in another limb.

### 4.4. Career Progression

Despite the persistence of low-grade lameness over serial examinations, many horses were still able to perform and increase their level of training and competition performance, with 61% reaching Prix St Georges level or higher. Every athlete deals with injuries that cannot be cured, such as osteoarthritis, or develops biomechanically inferior repair tissue with the inherent risk of reinjury; therefore, it is common that injuries accumulate with time. Those horses competing under FEI rules would have had to pass a horse inspection, assessed by the President of the Ground Jury and the Veterinary Delegate as fit to perform. Ideally, management and training programmes should be tailored to minimize the progression of injuries and to give ample recovery time between intense episodes of work. Work programmes should include cross-training to optimize proprioceptive function and to reduce the risk of repetitive strain injuries. It is advised that during warm-up prior to a competition, the aim is to mobilize a horse in as stress-free a manner as possible, to avoid repetitive overload and muscle fatigue, to minimize injury risk, and to optimize performance.

### 4.5. Metacarpal and Metatarsal Region Pain

There was a high prevalence of metacarpal and metatarsal region pain, predominantly due to suspensory desmitis, which was associated with a high rate of injury recurrence. Suspensory ligament injuries are common in dressage, showjumping, eventing, and general-purpose riding horses [10]. However, several studies have shown that dressage horses may be at greater risk of hindlimb suspensory desmopathy compared with horses in other work disciplines [3,10,23,72], although in another recent study, the prevalence in general-purpose riding horses had increased [73].

Proximal suspensory desmitis in forelimbs is often initially an acute overload injury, whereas hindlimb proximal suspensory desmopathy is probably a repetitive strain injury superimposed upon degenerative changes. Selection for advanced diagonal placement, increased extension of the metatarsophalangeal joints in collected and extended paces, and the biomechanical demands of piaffe, passage, and canter pirouettes, and repetitive work on artificial surfaces may all be contributory factors to the frequency of hindlimb proximal suspensory desmopathy in dressage horses [74,75].

#### Management and Prevention of Suspensory Ligament Injury

Further understanding about the role of cross-training as a preventative and management tool is required. Training techniques should be slowly progressive, with an emphasis on developing the correct muscle groups of the cervicothoracolumbar regions, the thoracic girdle, and the pelvis [76,77]. It is also recommended for young horses that excessive work in circles and extended trot should be limited because these are common scenarios for a first episode of suspensory desmitis [78]. There should be ample time for low-grade injury repair and progressive adaptation to load, as recommended in human sports medicine [79].

Hypermetric gaits may also affect the degree of fetlock extension and therefore the risk of suspensory ligament injury; further investigation comparing gait characteristics and the incidence of suspensory desmitis is merited. It appears that some horses have an innate susceptibility to suspensory ligament injuries [73], and the role of genetics and epigenetics deserves further investigation in Warmblood and Iberian horses. Excessive body condition score is a risk factor for injury [73], and dietary management should be considered as a preventative measure and during rehabilitation after injury. Maintenance of correct hoof balance and orientation of the distal phalanges by appropriate trimming and shoeing is also important [80].

### 4.6. Metacarpophalangeal or Metatarsophalangeal Joint Region Pain

There was a high prevalence of metacarpophalangeal or metatarsophalangeal joint region pain, most frequently associated with osteoarthritis or suspensory branch desmitis. In a Swedish study based on insurance company data of 107,310 horses, osteoarthritis of a metacarpophalangeal or metatarsophalangeal joint was the single most common cause of lameness, with a substantially higher incidence in Warmbloods compared with Thoroughbreds, Standardbreds, Ponies, Icelandic horses, and Coldblood horses [81]. Subchondral bone injury of the distal aspect of the third metacarpal bone and the proximal aspect of the proximal phalanx is increasingly being recognized as a cause of pain and lameness in sports horses, diagnosed using MRI [82,83,84,85] or CT [86,87]; however, showjumping horses have largely predominated, with dressage horses ranging from 7% to 50% of the reported groups. In dressage horses with persistent pain associated with osteoarthritis, despite intra-articular medication, it is suggested that examination using CT or MRI may be beneficial to better understand the nature of the injury.

In the current study, suspensory branch injuries were common. In a previous study, 46% of 71 horses with suspensory branch injuries were Warmbloods, although there was a similar prevalence of injury among dressage, eventing, showjumping, and general-purpose riding horses [88].

#### Management of Metacarpophalangeal or Metatarsophalangeal Joint Region Pain

In the current study, there was a high rate of persistent (osteoarthritis) or recurrent (suspensory branch desmitis) injury and lameness associated with metacarpophalangeal or metatarsophalangeal joint region pain. Careful timing of palliative intra-articular medication and monitoring of the suspensory branches by clinical assessment, and ultrasonography (including Power Doppler) are important for management, in addition to optimizing trimming and shoeing and the selection and maintenance of appropriate work surfaces [3,11].

### 4.7. Hypermetria and Weakness

Forty-three percent of horses were described as hypermetric at the initial examination, and this did not change over time. These horses may have been preselected as having the potential to be highly marked by judges. Modern Warmblood dressage horses have been bred to display gaits in which the antebrachial region of each forelimb is often raised higher than the crus of each hindlimb (so-called ‘leg movers’) [76]. In trot, advanced diagonal placement of the hindlimbs is rewarded by the judges despite the *FEI Dressage Handbook Guidelines for Judging* clearly stating that for working, collected, medium and extended trot there should be ‘Absolutely regular steps in clear two-time beat from beginning to end and with a clear moment of suspension’ [9]. Differentiation between a naturally high and long stepping gait with increased suspension and hypermetria due to neurological dysfunction can be challenging, and a comprehensive neurological evaluation should be performed. Iberian horses often display a different high-stepping forelimb and hindlimb gait, although in some horses this may be reduced if performance-limiting pain is removed by diagnostic anaesthesia [89]. A precise cause of hypermetria or hypermetria and weakness was not determined in the current study. Cerebellar, spinal cord, or upper motor neuron dysfunction should be considered.

#### Hypermetria, Weakness, and Performance

Horses displaying both hypermetria and weakness were more likely to have a poor outcome compared with other horses. Although horses with hypermetria and weakness were less likely to reach Prix St Georges level or above compared with both hypermetric horses and those with a neurologically normal gait, only 47% of hypermetric horses reached Prix St Georges compared with 81% of horses with a neurologically normal gait. This difference may be of biological significance. With hypermetria and weakness, it is possible that some horses did not develop sufficient musculoskeletal strength and coordination to consistently perform the more complex movements required for elite dressage. The relationship between hypermetria or hypermetria and weakness and the subsequent development of lameness merits further investigation.

### 4.8. Paddock Turnout

Access to paddock turnout did not have a significant effect on outcome, with a higher proportion of horses with no turnout having a good outcome compared with horses with turnout. In a previous study of risk factors for lameness in dressage horses, turnout was protective in the univariable analysis but was not included in the final multivariable model [3]. In a study of showjumpers, turnout was not a significant variable in the outcome ‘days lost to training’ when the most common reason for failure to train was lameness [67]. Nonetheless, there are many potential benefits of turnout in a paddock of adequate size: continual movement, lowering of the head and neck to graze with flexion of the thoracolumbar region, irregular terrain stimulating proprioceptive function, enhanced respiratory health, mental relaxation, and potential social interaction with conspecifics.

### 4.9. Dressage and the Social Licence to Operate

Dressage is under the spotlight with respect to the social licence to operate [90,91,92,93]. Nonetheless, it has previously been shown that the incidence of overt lameness in horses competing in Grand Prix dressage is low, which is reflected by low RHpE scores [94,95,96]. However, the display of conflict behaviors is high [94,95,96,97], and the reasons for this need to be addressed. Regular veterinary monitoring, including ridden horse assessment and evaluation of behavior using the RHpE, is therefore considered essential to recognize problems and treat accordingly, to improve comfort and trainability, reduce mistakes, prevent fatigue, and thus improve the scores obtained at competitions. Moreover, by reducing the occurrence of conflict behaviors, the public perception of the sport may improve.

### 4.10. Limitations of the Study

Like most clinically based and retrospective studies, the current investigation had limitations. Most of the study was performed in Spain, and therefore, there was a high proportion of Iberian horses, so the results may not be transferable to other populations of dressage horses in which Warmblood breeds predominate. However, the study included horses that ultimately competed internationally. The sample size was small. All clinical examinations were performed by a single clinician who may have a bias in their observations, including the interpretation of the responses to diagnostic anaesthesia, methods of investigation, and management strategies, and the methodologies used may also have evolved over time. Objective gait analysis may have added valuable information, but it was not available. However, it has some limitations with respect to the evaluation of ridden horses, evaluation of horses with multilimb lameness, the presence of neurological variations in gait, and abnormalities of canter. The riding and training techniques, general horse management, available footing, opportunities for cross-training, standards of trimming and shoeing, and tack fit are all factors that may influence injury risk and outcome. These factors were not evaluated in the current study. Potential biases of the owner, trainer, and rider may have affected their perceptions about performance and their decisions regarding the level of physical activity, participation in competitions, and retirement. Although 64% of horses had a good outcome and 61% reached Prix St Georges level or above, the quality of their performances was not assessed.

## 5. Conclusions

In this study of 70 dressage horses monitored longitudinally, the prevalence of lameness was high at the initial examination during the orthopaedic examination, although this was often not previously recognized. Despite diagnosis and management, most horses had ongoing problems, either with persistent or recurrent injuries or the development of additional orthopaedic injuries. Nonetheless, although only 3% of horses were competing at Intermediate I or II, U25, or Grand Prix at the initial examination, this increased to 37% by the final examination. Horses with lameness grade 0 or 1 at the initial examination had the best long-term outcomes. Regular monitoring, accurate diagnosis, targeted treatment, and appropriate management may enable dressage horses to fulfil their athletic potential and compete at the highest level despite orthopaedic problems.

## Figures and Tables

**Table 1:** Difference in the proportion of forelimb and hindlimb lameness in 70 dressage horses examined sequentially at six-months’ intervals for five examinations. N = number of limbs; % = percentage.

Examination Number	Left Forelimb Lameness N (%)	Right Forelimb Lameness N (%)	Two-Sample Test for Equality of Proportions *p*-Value
1	28 (40.0%)	22 (31.4%)	0.290
2	24 (34.3%)	29 (41.4%)	0.219
3	28 (40.0%)	21 (30.0%)	0.215
4	22 (31.4%)	20 (28.6%)	0.712
5	19 (27.1%)	21 (30.0%)	0.708
**Examination Number**	**Left Hindlimb Lameness** **N (%)**	**Right Hindlimb Lameness** **N (%)**	**Two-Sample Test for Equality of Proportions *p*-Value**
1	11 (15.7%)	11 (15.7%)	1.00
2	7 (10.0%)	8 (11.4%)	0.785
3	7 (10.0%)	13 (18.6%)	0.147
4	12 (17.1%)	11 (15.7%)	0.820
5	9 (12.9%)	10 (14.3%)	0.805

## Data Availability

Anonymized data are available from the authors upon reasonable request.
